# When Targeted Therapy Falls Short: Unraveling Resistance Mechanisms in Chronic Lymphocytic Leukemia

**DOI:** 10.1007/s11899-026-00776-3

**Published:** 2026-02-26

**Authors:** Samon Benrashid, Jennifer A. Woyach

**Affiliations:** The Ohio State University Comprehensive Cancer Center, 410 W 10th Ave, Columbus, OH 43210 USA

**Keywords:** Chronic lymphocytic leukemia, Targeted therapy, Bruton’s tyrosine kinase inhibitors, BTK mutations, Bcl-2, Venetoclax

## Abstract

**Purpose of Review:**

Targeted therapies have revolutionized the treatment of chronic lymphocytic leukemia (CLL), however the disease remains incurable. This is largely due to somatic mutations in proteins targeted by these therapies, like Bruton’s tyrosine kinase (BTK) and B cell lymphoma-2 (Bcl-2), or transcriptional rewiring of CLL cells. Here we review recent findings regarding mechanisms of resistance to targeted agents in CLL.

**Recent Findings:**

BTK inhibitor (BTKi) resistant CLL is potentiated by mutations disrupting covalent and non-covalent BTKi binding or those conferring loss of BTK’s kinase activity. Point mutations in Bcl-2 impact the efficacy of Bcl-2 inhibitor venetoclax, however cells employ a diverse variety of mechanisms to escape venetoclax-induced apoptosis. These mechanisms function through AKT and result in increased dependency on and stabilization of other antiapoptotic Bcl-2 family members as well as disruption of BAK/BAX pore formation.

**Summary:**

Recent work in CLL has pinpointed mechanisms hijacked by cells to abrogate treatment efficacy. Ongoing research efforts are focused on the advent of next-generation inhibitors and protein degraders to circumvent resistance. Such studies will prove invaluable in providing CLL patients with a diverse repertoire of therapeutic options following relapse.

## Introduction

Chronic lymphocytic leukemia (CLL) is the most common adult leukemia in western countries and is characterized by a clonal expansion of immunologically dysfunctional CD19 CD5 dual-positive B cells within the blood, bone marrow, spleen, and lymph nodes [1]. Traditionally, clinical outcomes have been markedly variable in CLL and largely related to specific molecular features associated with the disease [2]. Patient cohorts often display significant heterogeneity in these molecular features, with putative drivers presenting as somatic copy number alterations (sCNAs) rather than translocations pertaining to the Ig loci [3]. Yet, irrespective of specific molecular features, CLL remains incurable for most patients and there is a lack of therapeutic options following relapse on contemporary treatments [4, 5]. 

Antigen-dependent or -independent activation of the B cell receptor (BCR) serves a critical role in CLL cell proliferation and survival, and as such represents a potential therapeutic vulnerability. Indeed, targeting the BCR pathway has proven fruitful in CLL and over the last two decades we have witnessed a paradigm shift away from traditional chemoimmunotherapy towards small molecule inhibitors. Therapeutic agents targeting Bruton’s tyrosine kinase (BTK) have proved efficacious at interrupting BCR signaling and are now an invaluable treatment strategy for CLL, providing durable remission periods up to 10 years. To date, four BTK inhibitors (BTKi) have received FDA approval for use in CLL (ibrutinib, acalabrutinib, zanubrutinib, pirtobrutinib). Yet, given enough time on BTKi patients invariably relapse, typically through acquired point mutations in *BTK* which reduce BTKi binding affinity.

Inhibition of the anti-apoptotic protein B cell lymphoma-2 (Bcl-2) using venetoclax has also become a frontline option in CLL, demonstrating tremendous efficacy [5–7]. By displacing pro-apoptotic proteins sequestered within the hydrophobic binding pocket of Bcl-2, effector and executioner molecules, like BAK and BAX, are released to increase mitochondrial outer membrane permeabilization (MOMP). This process ultimately results in cytochrome c release, caspase activation, and intrinsic apoptosis [8]. Though, the potency of VEN can be limited by point mutations in *BCL2* acquired over a treatment course. The same may be said for other cases where we observe a shift away from Bcl-2 reliance to other anti-apoptotic Bcl-2 family members like Mcl-1 or Bcl-xL. Thus, even following the evolution of treatment regimens we remain face to face with the inevitable challenge of therapeutic resistance. In this review, we will evaluate current and recently completed work regarding molecular mechanisms of resistance to BTKi and VEN in CLL and other B cell malignancies, as well as potential strategies to overcome these resistances.

## BTKi in CLL Overview

### BTK

BTK is a cytoplasmic protein tyrosine kinase within the Tec family of kinases that is essential for BCR signaling. Following BCR stimulation, BTK is phosphorylated by LYN/SYK allowing induction of calcium ion release as well as BTK’s downstream kinase function for propagating differentiation, pro-survival, and pro-growth signals via signal transduction. CLL is characterized by constitutive autonomous BCR signaling regardless of antigen stimulation.

Preceding the emergence of BTKi for therapeutic use, preclinical experiments demonstrated both genomic inactivation and pharmacologic inhibition of BTK were effective in limiting CLL cell growth and survival [9, 10]. These initial studies identifying BTK as a potential therapeutic target have been greatly rewarding and so far four BTK inhibitors have been approved by the FDA for use in CLL. These are comprised by covalent BTK inhibitors (cBTKi) ibrutinib, acalabrutinib, and zanubrutinib, as well as the non-covalent BTK inhibitor (ncBTKi) pirtobrutinib. Notably, successive generations of cBTKi have capitalized on increased specificity towards BTK and reduced off-target potency compared to ibrutinib [11]. Pirtobrutinib has also shown a favorable adverse events profile compared to all cBTKi [12].

Clinically, all three cBTKi have demonstrated efficacy in treatment-naïve CLL [11, 13–18]. The first head-to-head study of cBTKi, ELEVATE-RR, showed non-inferiority of acalabrutinib in comparison to ibrutinib, with acalabrutinib demonstrating a better safety profile [19]. The ALPINE study made a similar comparison between zanubrutinib and ibrutinib in relapsed or refractory (R/R) CLL and demonstrated superiority in overall response when comparing zanubrutinib to ibrutinib [15]. Superiority of zanubrutinib for both complete response and long-term tolerability were also observed in Waldenström macroglobulinemia by the ASPEN study [20, 21]. Yet, even with improved outcomes in these patient populations progression free survival (PFS) is ultimately limited by resistance-associated mutation acquisition.

### Resistance To BTKi

**Fig. 1:**
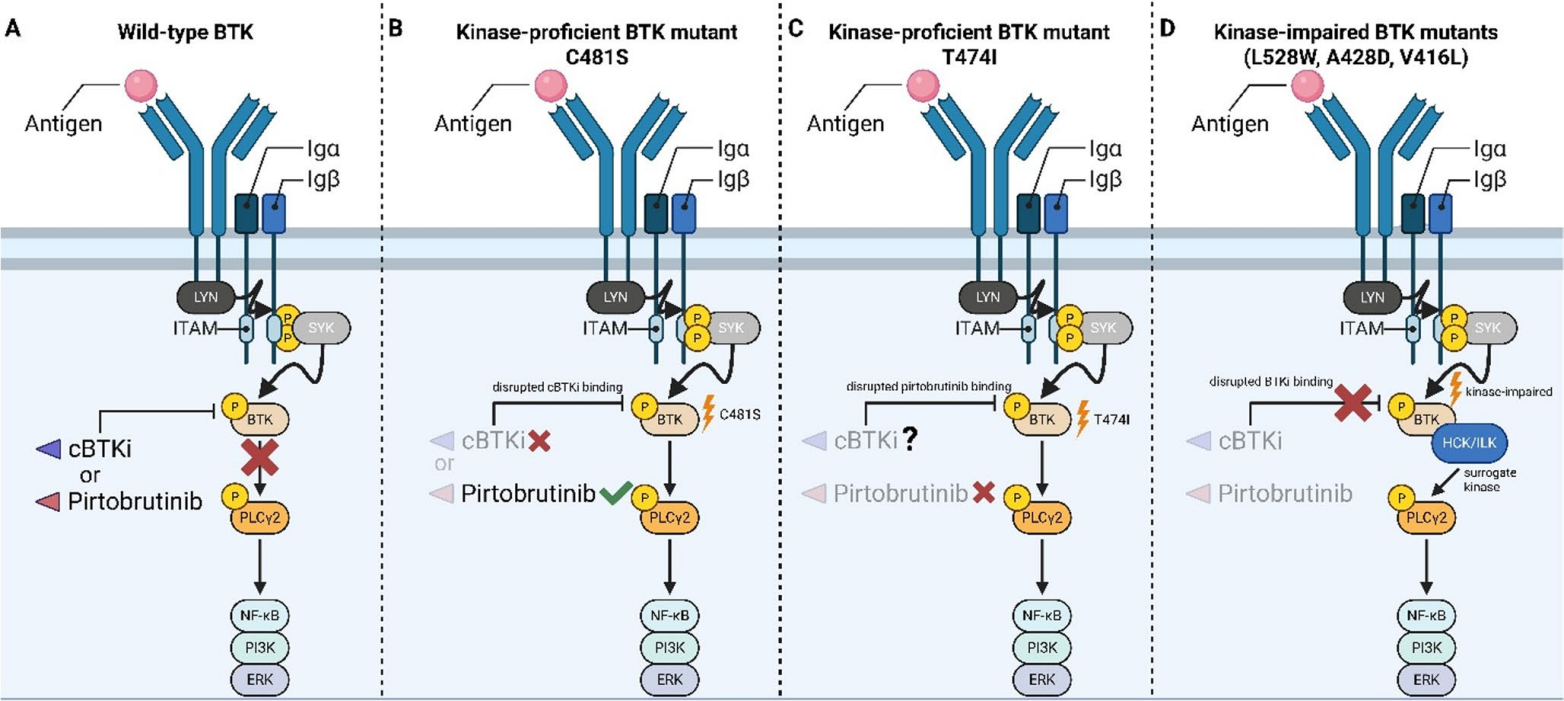
Mechanisms of resistance to BTKi. (**A**) In the setting of wild-type BTK, both cBTKi and ncBTKi are efficacious at shutting down active BCR signaling. (**B**) Following cBTKi, emergent C481S mutations disrupt the ability of cBTKi to interact covalently with BTK and BCR signaling remains intact. In this setting, administered ncBTKi bypasses the need of an unchanged C481 residue and effectively silences signaling through the BCR. (**C**) Resistance to ncBTKi may arise through gatekeeper mutations at the T474 residue and permit BCR signaling. Future studies will evaluate administration of cBTKi following resistant to frontline ncBTKi therapy. (**D**) Kinase-impaired BTK mutants demonstrate resistance and downstream BCR signal persistence irrespective of administered BTKi

First- and second-generation BTK inhibitors work to functionally silence BTK activity via irreversible (covalent) binding to the cysteine 481 (C481) residue, and prior to treatment with targeted therapeutics BTK mutations are not a recurrent feature of CLL (Fig. 1A) [22, 23].

Due to the importance of BTK in CLL biology and the selectivity of BTK-targeting agents, mutations in BTK have been shown to be the most common mechanism of resistance to date [24]. Here, we will discuss various BTK mutations which confer acquired resistance to cBTKi and ncBTKi. Though, it is also of important note that BTKi-resistant CLL can arise through non-BTK mediated adaptations activating pro-growth and pro-survival pathways in CLL cells, typically NF-κB, PI3K, and MAPK [25, 26].

### Kinase-proficient BTK Mutations (C481, T474)

Following the clinical implementation of ibrutinib, and subsequent generations of cBTKi, it was reported that CLL patients relapsing during cBTKi therapy present with missense mutations affecting BTK’s C481 residue (> 90% of BTK mutations). Most notable is a cysteine-to-serine change (C481S) resulting in retained enzymatic activity and abrogation of irreversible cBTKi binding [22]. Instead, cBTKi interact reversibly (non-covalently) with BTK in the presence of C481 mutations, limiting their potency at shutting down BCR signaling due to the short half-lives of these agents (Fig. 1B). Reports from recently completed phase III clinical trials comparing first- and second-generation cBTKi in the R/R setting suggest the vast majority of patients relapsing on treatment present with missense mutations at C481 [27]. Importantly, there do not appear to be major differences in C481 mutation rates when comparing ibrutinib versus acalabrutinib therapy (53% vs. 51%, respectively) in patients experiencing progressive disease (PD) [25].

The arrival of cBTKi resistance necessitated work in the development of therapeutic interventions targeting BTK covalently at other residues or in a non-covalent (reversible) fashion. Early development and investigation into ncBTKi yielded promising results and to date we have seen the clinical emergence of a few ncBTKi, with nemtabrutinib and pirtobrutinib serving as the two most clinically developed ncBTKi [12, 28–32]. Functionally, these inhibitors rely on weaker, more transient interactions like hydrogen bonds, ionic bonds, and hydrophobic interactions to facilitate their inhibitory properties [33]. This allows them to functionally inhibit C481-mutant BTK by mitigating reliance on the presence of C481 and rather exert their effect by targeting BTK’s gatekeeper residue, threonine 474 (T474) [34, 35].

Preclinically, nemtabrutinib and pirtobrutinib demonstrated similar efficacies in the presence of wild-type (WT) and C481-mutant BTK whilst boasting a longer half-life (≥ 20 h) when compared to cBTKi [29, 31]. Early clinical studies using these drugs in R/R CLL have shown them to possess high activity following cBTKi discontinuation or resistance. Nemtabrutinib demonstrated a median PFS in phase 1 trials, with definitive trials ongoing. Pirtobrutinib, which has received accelerated approval by the US FDA, has a median PFS of 14–19 months [28, 30, 36, 37]. Importantly, there are no observed decreases in ncBTKi efficacy when comparing their use in patients harboring C481 BTK mutations to those who are not [28, 38].

Mutations at the T474 residue had been reported following discontinuation of ibrutinib and acalabrutinib, but are significantly enriched in patients treated with ncBTKi [24, 39]. Most frequently observed is a threonine-to-isoleucine (T474I) mutation at this site, which was predicted early on to function as a gatekeeper mutation targeting a critical residue for ncBTKi binding using in vitro systems [35]. Following this, clinical mechanisms of ncBTKi resistance were reported in two pirtobrutinib-treated cohorts, with a majority of patients acquiring a new, non-C481 mutation in BTK’s kinase domain [24, 36]. Indeed, T474 mutations were enriched in these cohorts and allowed BTK to retain its kinase activity, suggesting a major role in clinical resistance mechanisms following exposure to ncBTKi (Fig. 1C) [24, 35, 36].

### Kinase-impaired BTK Mutations (L528W, C481F/R/Y, M437R, A428D, V416L)

Until 2019 there was a lack of observed kinase-impaired mutations in patients treated with BTKi, limiting prior knowledge of these mutations [40]. However, reports in CLL over the last six years have increasingly identified BTK mutations conferring a variant of the protein unable to promote autophosphorylation at Y223 due to mutations targeting key residues in BTK’s ATP-binding interface (L528, A428, V416) [24, 41, 42]. Paradoxically, clones with kinase-impaired mutants retain BCR signaling as NF-κB, PI3K, and MAPK activities persist irrespective of BTKi treatment [24, 43]. This phenomenon has been posited to a secondary scaffold function retained in kinase-impaired BTK mutants wherein mutant BTK acts as a membrane backbone to recruit other kinases such as hematopoietic cell kinase (HCK) and integrin-linked kinase (ILK) (Fig. 1D). Thus, following their recruitment, these proteins are activated and promote phosphorylation of PLCγ2 to propagate BCR signaling [43, 44].

Initially these mutations were unexpected altogether, as it was commonly believed they would functionally self-abrogate BCR signaling by disrupting BTK’s ATP binding and subsequent enzymatic function [44]. But reports of kinase-impaired mutations have become more abundant since their discovery. This has also coincided with an ever-changing paradigm in the BTKi treatment landscape, suggesting that BTK resistance mutations may emerge differentially in a BTKi-specific manner. This phenomenon was first observed in zanubrutinib treated patients where a treatment-resistant clone bearing a leucine to tryptophan mutation at BTK’s L528 residue (L528W) was observed in 7 of 13 patients progressing on zanubrutinib, but only 1 of 24 progressing on ibrutinib [42]. This trend was also observed in patients progressing on the cBTKi tirabrutinib, and at a higher rate than study participants taking ibrutinib or acalabrutinib [45]. Interestingly, kinase-impaired L528W mutants are also enriched in CLL patients progressing on pirtobrutinib, suggesting this as a potential mechanism for escape from certain c- and ncBTKi [24, 46]. Kinase-impairment to BTK can also arise from mutations at residue A428, commonly A428D, and V416, commonly V416L. Though initial discovery of these mutants came when evaluating mechanisms of ncBTKi resistance, in vitro experiments suggest A428D limits the efficacy of all FDA approved cBTKi while V416L may still respond to zanubrutinib [24, 47, 48]. Moreover, the efficacy of BTK degraders, previously thought to be unhindered by kinase-domain BTK mutations, may also be abrogated by A428D mutations [49]. Though, given the relative infancy of BTK degraders, it will take further studies to fully understand emergent patterns of resistance.

It is important to note there are likely major differences in the specific mechanisms through which kinase-impaired BTK mutants promote BCR signaling, as these mutations typically broaden the range of BTK’s possible protein-protein interactions [44]. For example, one might hypothesize that treatment with a more promiscuous BTK inhibitor targeting HCK, such as ibrutinib or nemtabrutinib, could functionally silence BCR signaling in these mutants [31, 50]. However, preclinical studies using modified cell line variants suggest cells with kinase-impaired BTK mutations (L528W, C481F, M437R, A428D, V416L) are more resistant to both compounds [24, 47]. This idea is also supported by clinical observations regarding the emergence of C481F/R/Y kinase-impaired BTK mutants following exposure to ibrutinib [27, 51–53].

Given the relative complexity shaping resistance in kinase-impaired BTK mutants, more work is needed to elucidate a comprehensive catalogue of mechanistic adaptations promoting disease progression in patients with these mutations [33, 51].

## Bcl-2i in CLL Overview

### Bcl-2

Evasion of apoptosis is a hallmark of cancer, and this feature is not lost in CLL [54–57]. Typically, successful apoptosis is triggered by increased MOMP resulting from BH3 protein-mediated displacement of apoptotic executioner molecules (BAK/BAX) sequestered within Bcl-2’s hydrophobic pocket, allowing homo- or heterodimerization in the mitochondrial outer membrane and subsequent release of cytochrome *c*, a process known as the intrinsic mechanism of cell death [8]. CLL cells effectively avoid this process by increasing expression of anti-apoptotic proteins, most notably Bcl-2, in addition to downregulating pro-apoptotic executioners, to suspend regulated cell death in favor of survival [58–60]. As such, these anti-apoptotic proteins have emerged as therapeutic targets over the last two decades.

Early studies evaluating the small-molecule navitoclax, designed to inhibit Bcl-2 as well as its anti-apoptotic family member Bcl-xL, in patients with R/R CLL showed efficacy with a median PFS of 25 months. However, development in CLL was limited due to on-target platelet toxicity mediated through inhibition of BCL-xL [61]. This observation led to the development of the platelet sparing, Bcl-2-specific inhibitor venetoclax (VEN) which is now approved for treatment of CLL [62, 63]. To date, multiple studies have shown VEN efficacy in R/R and treatment-naïve settings of CLL, typically in combination with an anti-CD20 monoclonal antibody, where there are observed remission periods associated with undetectable minimal residual disease (MRD) [5, 6, 64–66]. VEN has also been studied in combination with BTKi, where doublets of BTKi plus VEN, or triplets of BTKi plus VEN plus obinutuzumab have been demonstrated to be effective in frontline CLL [67, 68]. The GLOW study combined ibrutinib and VEN for a 1-year fixed duration to investigate this and reported a 42-month PFS rate of 74.6% [67]. Using a triplet regimen, the AMPLIFY study reported an 83.1% PFS at 3-years following 1-year fixed duration acalabrutinib, VEN, and obinutuzumab [68].

VEN regimens are commonly used following resistance to BTKi, though the benefits/consequences to differential sequencing of therapies are unclear at the moment, as our group has recently discussed [16]. Unlike BTKi, patient outcomes following VEN treatment are more likely to be negatively impacted given higher patient risk scores (Unmutated *IGHV*, *TP53* alteration). And, despite the tremendous success VEN has shown in CLL, therapeutic resistance still arises, typically following long-term treatment or in the context of treatment for R/R patients [69, 70].

### MRD

Tracking MRD is a largely useful tool in CLL to gain insight into the current and future efficacy of a given therapy. Multiple clinical trials have demonstrated the ability of VEN to induce undetectable MRD (uMRD) primarily using cutoffs of 1 CLL cell in 10,000 leukocytes, which has been especially successful in the frontline setting [6, 71]. Long-term data demonstrates that patients who achieve uMRD have longer remission durations compared with those who have detectable MRD at the end of therapy (61.5% vs. 22.7% 6-year PFS, respectively) [66]. In VEN combinations with BTKi, the predictive value of MRD appears to be more significant in patients with IGHV unmutated disease.

MRD evaluation has been proposed as a mechanism to determine duration of therapy. This has been studied in the context of both doublets and triplets. The FLAIR study demonstrated the feasibility of an MRD-guided treatment approach in the setting of ibrutinib plus VEN, where MRD was used to determine therapy length. Here, 56.3% of patients discontinued therapy by 3 years and 68.1% by 4 years. Long-term PFS following MRD-guided ibrutinib plus VEN has been outstanding with 97.2% PFS at 3 years and 93.9% at 5 years [72–74]. Multiple ongoing clinical trials are evaluating the efficacy of MRDguided approaches for treatment in the context of second generation BTKi.

### Resistance To Bcl-2i

#### *BCL2* Gene Mutations

Resistance to VEN in CLL via point mutations within the hydrophobic binding pocket of Bcl-2 are common, accounting for nearly 40–50% of patients experiencing PD, with glycine-to-valine (G101V) and aspartate-to-tyrosine (D103Y) being the most prevalent mutations mediating resistance [69, 70, 75–77]. These mutations are reported to diminish VEN binding affinity for Bcl-2 by disrupting its ability to form a hydrogen bond critical for efficacy [62]. Meanwhile, Bcl-2’s affinity for sequestering pro-apoptotic executioners and its anti-apoptotic capabilities remain intact [76, 78]. Interestingly, mutations at A113 and V156 have also been reported, though these tended to cooccur with G101V or D103Y within a patient’s tumor cell population [75, 79].

### Regulation of Other Bcl-2 Family Members

**Fig. 2:**
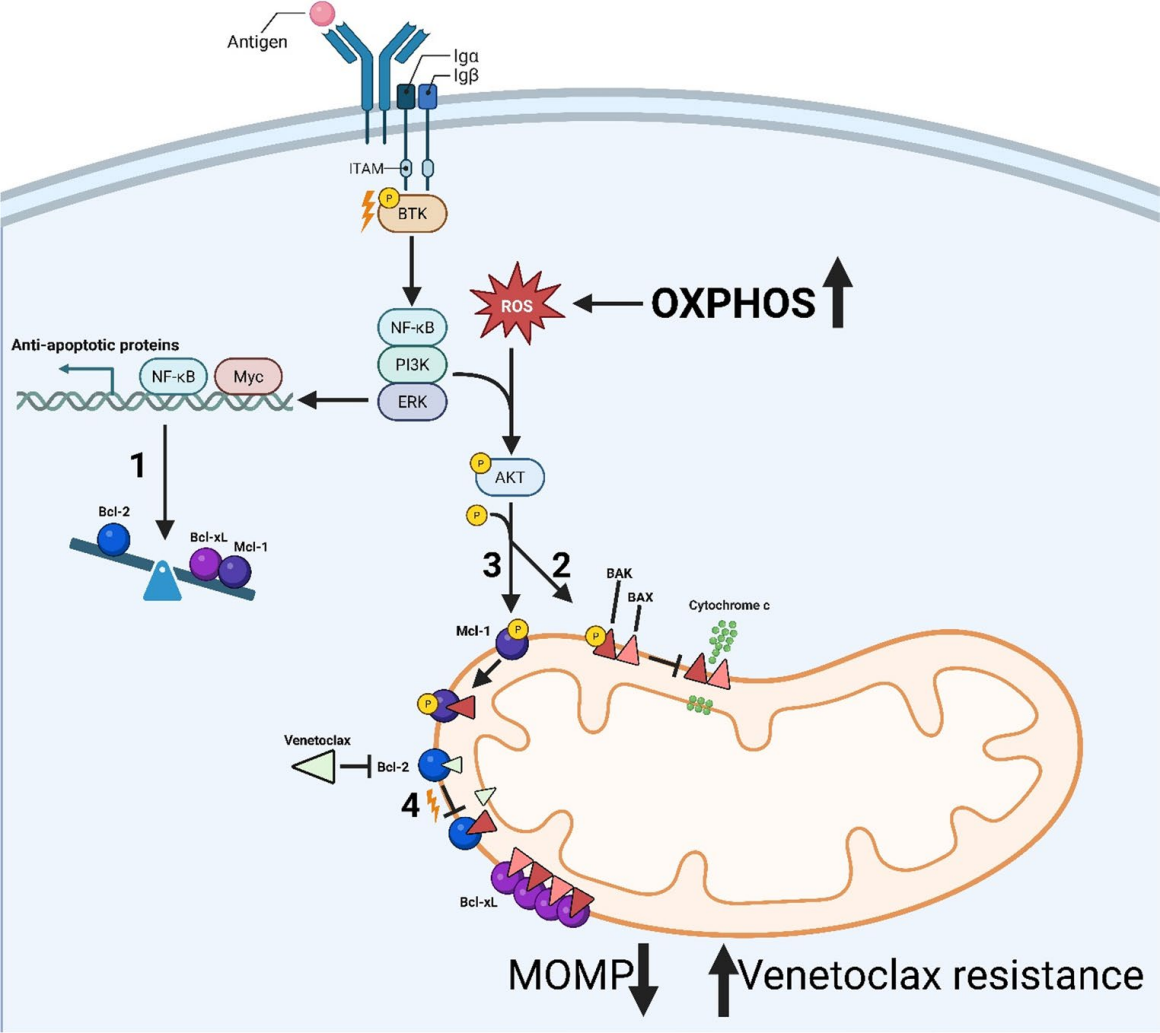
Mechanisms of resistance to VEN. It has been previously reported that resistance to VEN can result from transcriptional reprogramming and shifted dependencies on antiapoptotic proteins, rendering inhibition of Bcl-2 less impactful (1). Resistance may also come about via AKT-mediated phosphorylation and inactivation of pore-forming proteins like BAK/BAX, inhibiting the release of cytochrome c into the cytosol (2). Additionally, reactive oxygen species (ROS) can mediate AKT activation, resulting from heightened oxidative phosphorylation (Oxphos), leading to phosphorylation and stabilization of Mcl-1 protein (3). Lastly, Bcl-2 missense mutations affecting the hydrophobic binding pocket may disrupt VEN binding and allow Bcl-2 mediated sequestration of proapoptotic protein in the presence of VEN

The dynamics of how Bcl-2 point mutations manifest within resistant tumor cell populations are not entirely understood as patients typically present these mutations at a low variant allele frequency (VAF). And, as previously mentioned, resistance to VEN can be acquired through a plethora of mechanisms outside of Bcl-2 missense mutations, suggesting more than one mechanism is likely contributing to VEN resistance within any single patient (Fig. 2). For example, mutations or somatic copy number alterations (sCNAs) in BAX promote dysfunctional apoptotic signaling due to decreased protein expression or improper protein localization [80, 81]. Both loss(8p) and gain(1q) have been shown to result in Mcl-1 overexpression and VEN resistance in CLL, likely as a consequence of increased ERK signaling [82].

Reports in patients harboring *TP53* alteration or heightened NF-κB signaling suggest sub-clonal upregulation of anti-apoptotic family members Bcl-xL and Mcl-1, thereby decreasing their sensitivity to VEN [61, 83–88]. One possible mechanism for this is the c-MET/hepatocyte growth factor signal axis which promotes AKT/ERK/STAT3 activation and escape from VEN-induced apoptosis [89]. This may feed forward into metabolic rewiring of CLL cells towards oxidative phosphorylation (Oxphos) and decreased Bcl-2 reliance, both of which have recently been associated with poor prognostic factors [90]. Furthermore, superoxide formation resulting from increased Oxphos may stabilize Mcl-1 in CLL via increased AKT activation promoting Mcl-1 phosphorylation, stability, and BAX inactivation to promote VEN resistance [91, 92].

Lastly, epigenetic rewiring can have significant impacts on the clinical efficacy of VEN. Overexpression of Bcl-xL is not associated with sCNA gains in CLL, but rather enhancer modifications [79]. Increased methylation of the *PUMA* promoter, which encodes the pro-apoptotic BH3-only protein PUMA, and subsequently decreased protein expression have been associated with VEN resistance and increased glucose metabolism [81]. Once more, this gives credence to the idea that metabolic rewiring may underlie VEN resistance for a portion of CLL patients by stripping cells of their ability to prime for apoptosis.

### Next-Generation Molecules To Combat Resistance

#### Overcoming BTKi Resistance

Combating the common resistance mechanisms of cBTKi and ncBTKi is becoming increasingly important. Following primary-site BTK mutations at C481, and second-site, gatekeeper mutations at T474, BCR signaling remains intact. This suggests that alternative mechanisms to continue targeting the BCR may have clinical utility.

Kinase inhibitors which target BTK outside of the C481 and T474 residues are currently being evaluated in preclinical and clinical studies. One of these, rocbrutinib, has been shown in preclinical studies as well as early clinical trials (Table 1) to have efficacy in patients harboring BTK resistance mutations, including a subset of patients with T474 mutations [93, 94].

**Table 1:** Clinical trials of novel agents relevant to CLL

Treatment	Molecule Class	Protein Target	Disease	Administered Therapies	Clinical Trial
Rocbrutinib	Inhibitor	BTK	R/R B cell malignancies	Rocbrutinib	NCT04775745 (Phase I)
			Previously treated CLL/SLL with/without T474 mutation	Rocbrutinib in combination with obinutuzumab	NCT06978088 (Phase II)
BGB-16,673	Degrader	BTK	B cell malignancies	BGB-16,673	NCT05006716 (Phase I/II)
NX-5948	Degrader	BTK	R/R B cell malignancies	NX-5948	NCT05131022 (Phase I)
MS553	Inhibitor	PKCβ	CLL/SLL	MS553 monotherapy, or combination with either acalabrutinib or VEN, rituximab, and obinutuzumab	NCT03492125 (Phase I/II)
DT2216	Degrader	Bcl-xL	R/R solid tumors	DT2216	NCT04886622 (Phase I)
			R/R solid tumors	DT2216 in combination with standard of care	NCT06620302 (Phase I/II)
			Platinum-resistant ovarian cancer	DT2216 in combination with paclitaxel	NCT06964009 (Phase Ib)
Sonrotoclax	Inhibitor	Bcl-2	CLL/SLL/MCL	Sonrotoclax dose escalation in combination with zanubrutinib or rituximab	NCT06839053 (Phase II)
Lisaftoclax	Inhibitor	Bcl-2	Treatment naive CLL/SLL	Lisaftoclax in combination with acalabrutinib	NCT06319456 (Phase III)

In recent years, it has become increasingly popular to target proteins for degradation, rather than inhibition. Protein degraders function by hijacking the cell’s ubiquitin-proteasome machinery and recruiting it to oncogenic proteins, such as BTK, to induce their degradation [95]. Given BTK’s hypothesized functional role as a scaffolding protein, in conjunction with our lack of effective therapeutic options targeting BTK following C481 and T474 mutations, this is indeed an attractive therapeutic paradigm in 2026. And, critically, these degraders appear to remain agnostic to BTK, regardless of mutation [96]. Early data from clinical trials using BTK-degraders have been encouraging, thus far, with BGB-16,673 and NX5948 reporting response rates of 78% and 76% in patients who have received cBTKi and Bcl-2i, respectively [97, 98]. Significantly, however, early data suggests that resistance to these degraders will be mediated by new BTK mutations that may preclude additional BTK-targeting [99].

BTKi resistance may also be overcome by targeting alternative proteins in the BCR pathway, such as those distal to BTK. Preclinical and clinical studies have evaluated PKCβ as a therapeutic target. Clinical data is early, but suggests that this might be an effective target in CLL (Table 1) [100–102].

### Overcoming Bcl-2i Resistance

Given the recent popularity and emergence of BTK-degraders, it is of little surprise that there is also interest in molecules targeting antiapoptotic Bcl-2 family proteins for degradation. The early Bcl-2/Bcl-xL degrader DT2216, for example, possesses a navitoclax-based warhead and leverages an E3-ubiquitin ligase that is lowly expressed in thrombocytes, helping to spare platelet toxicity [103]. This first in human compound has recently completed phase I clinical trial in R/R solid malignancies (NCT04886622**)** and begun testing in a phase I/II trial (NCT06620302) (Table 1). More recent work to develop dual Bcl-2/Bcl-xL degraders resulted in WH244, a compound with more potent disruption of antiapoptotic signaling compared to previous generations. The work done with WH244 has also established a framework for structural and mechanistic studies of degraders, which will undoubtedly be useful as the field continues to move forward [104]. Overall, the use of degraders represents a promising upcoming paradigm. These molecules have the potential to make previously undruggable targets druggable, vastly expanding the potential therapeutic landscape for a number of malignancies. Additionally, it will be of great clinical use to limit off-tumor toxicities by leveraging specific E3 ligase expression across different tissues.

There is also great interest in the development of next-generation Bcl-2 inhibitors and multiple ongoing clinical trials in CLL (Table 1). Sonrotoclax and lisaftoclax are two such molecules and have demonstrated greater potency, compared to VEN, even inhibiting G101V mutant Bcl-2 [105–107]. Of note, sonrotoclax and lisaftoclax have demonstrated anti-leukemic activity and are both in phase III clinical trials as monotherapy or in combination with a second-generation cBTKi (NCT06839053 and NCT06319456) [108–110]. Though, currently it is unclear whether these next-generation inhibitors will simply replace VEN as first-line options for Bcl-2i or be used following VEN-resistance. Investigations utilizing BH3 profiling of CLL cells following treatment with these inhibitors may give insight into whether these agents promote similar resistance mechanisms as VEN or if they can be used as monotherapy in the VEN-resistant setting.

Either way, our knowledge of the current landscape of CLL resistance to VEN makes it likely that the use of degrader molecules or targeted therapies will need to be supplemented with small molecule inhibitors targeting antiapoptotic proteins other than Bcl-2. We have also learned, by use of BH3 profiling, that CLL cells are as sensitive to inhibition of these antiapoptotic family members as they are to inhibition of Bcl-2 itself [111]. So, while molecules like sonrotoclax and lisaftoclax retain some efficacy against common Bcl-2 missense mutations, their use will likely still be limited in VEN-resistant settings due to their lack of inhibiting other antiapoptotic Bcl-2 family proteins. Therefore, exploration of agents targeting BCL-xL or Mcl-1 is of high clinical interest.

Mcl-1 tends to be the other major player in VEN-resistant CLL, and as such small molecule inhibitors targeting Mcl-1 have been investigated in this setting. Notably, Mcl-1 contains a much broader hydrophobic binding pocket compared to Bcl-2 and Bcl-xL, making it more difficult to design appropriate BH3-mimetics for its inhibition [112]. Unfortunately, while a variety of Mcl-1 inhibitors have been published preclinically, few have made it into or beyond phase I clinical trials, mostly due to cardiac-related toxicities [113–117]. Should Mcl-1 be pursued further it will be necessary to minimize the risk associated with Mcl-1 inhibition by selectively targeting tumor populations. Thus, there is use in continued interrogation into the design and efficacy of tissue-specific Mcl-1 inhibition via other mechanisms, such as antibody-drug-conjugates or antibody degrader conjugates [118–121].

### Sequencing of Therapy in CLL

Currently, choice of therapy for relapsed CLL is dependent on the choice made for initial therapy, which would take into account genomic and clinical characteristics of the patient, as well as patient goals and preferences. No consensus exists on which paradigm should be chosen for frontline treatment (fixed duration vs. continuous), and it is generally accepted that for most patients it is appropriate to start with either continuous BTKi or fixed duration VEN-containing regimens and then utilize the other class at progression. With fixed duration regimens including VEN, option for retreatment exists, which may extend the use of a given class of drugs.

**Fig. 3:**
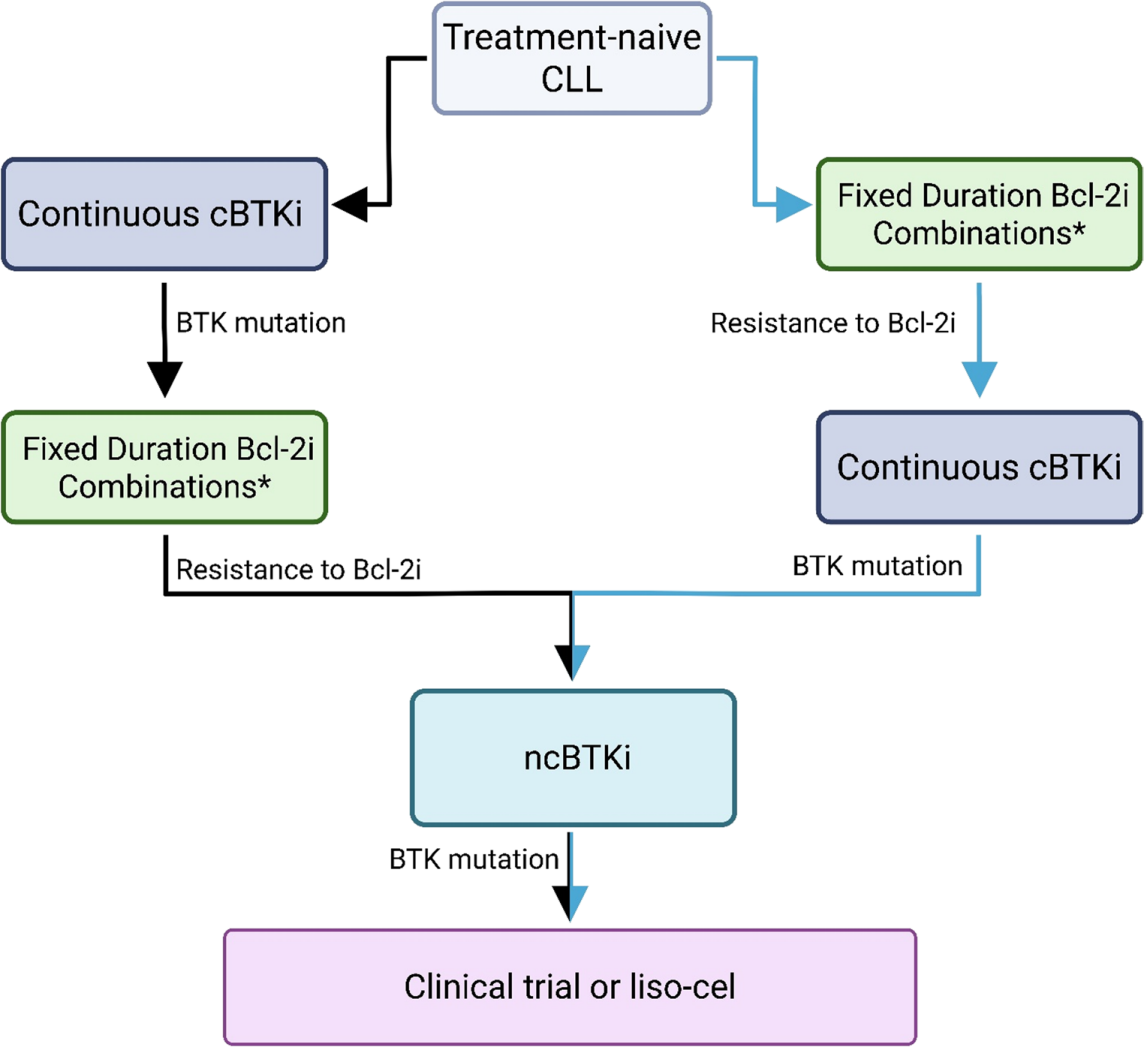
Therapeutic sequencing in CLL. Following frontline cBTKi, patients are likely to respond well to treatment with Bcl-2i, and vice versa. After discontinuation of secondary therapy due to progressive disease, ncBTKi serve as strong tools for combatting CLL. Following relapse, clinicians may opt for diagnostic sequencing to determine the optimal protein target and therapeutic avenues. *Fixed duration Bcl-2i combinations can be repeated

As outlined in Fig. 3, knowledge of resistance mechanisms and mutation acquisition can guide therapeutic sequencing. Utilizing only approved therapies, for patients who begin therapy with a cBTKi, presence of a C481 mutation in BTK at relapse would predict efficacy with either a ncBTKi or VEN, while presence of a mutation in PLCG2 or a gatekeeper or kinase-impaired mutation in BTK would steer a clinician toward VEN. For patients who begin treatment with VEN, retreatment could be considered in the setting of long remission duration (at least 3 years). Once retreatment is exhausted, utilization of BTKi pathway, liso cel, or currently available clinical trials would be recommended.

### Novel Strategies and Targets

Following relapse from standard BTKi and VEN therapies, several other options exist, both on our outside of clinical trials. Chimeric antigen receptor T cell (CAR-T) therapy has received accelerated approval in CLL and could be considered for fit patients who have received at least 2 lines of therapy.

A number of early and late-stage clinical trials are investigating novel targets and mechanisms in CLL. BTK degraders, as previously discussed, represent a promising therapeutic option for patients whose CLL has relapsed in the setting of cBTKi and ncBTKi. Alternative targeting of the BCR pathway is under investigation, including through inhibition of downstream targets such as PKCβ [100, 122]. For patients harboring Bcl-2 point mutations, treatment with novel small molecule inhibitors or degraders targeting Bcl-2 and/or its anti-apoptotic family members may be beneficial to overcome therapeutic resistance. Lastly, clinical implementation of immunotherapies, including chimeric antigen receptor T cell (CAR-T) and bi-specific antibodies (biAb) targeting CD19/CD20 and CD3, are active irrespective of resistance mutations and can be used in double or triple exposed patients.

## Conclusion

As the treatment paradigm for CLL has swung towards targeted therapies over the last two decades, it is safe to say we find ourselves in the golden age of research and development in this frontier. Still, current options for CLL treatment are not curative, and CLL patients invariably relapse while on treatment for this reason. Herein, we have discussed these major hurdles in the effective treatment and management of CLL following relapse on both BTKi and Bcl-2i, where patients have limited options. We believe the advent and use of agents targeting BTK or antiapoptotic Bcl-2 proteins in a novel fashion, which has progressed rapidly over the last two decades, represents the future of CLL treatment. Degrader molecules targeting BTK are undergoing preclinical and clinical investigation in CLL to evaluate their safety and efficacy. The same can be said for next-generation BTK and Bcl-2 inhibitors, which have thus far shown promising results as monotherapies or in combination with currently approved inhibitors. There are also ongoing studies aimed at designing therapies targeting antiapoptotic proteins other than Bcl-2, as many patients in the VEN-resistant setting will shift their reliance in favor of these other proteins. Continued research into novel therapies in CLL is essential to continue our positive momentum in this disease and work for a cure.

### Key References

- Wang E, Mi X, Thompson MC, Montoya S, Notti RQ, Afaghani J, et al. Mechanisms of Resistance to Noncovalent Bruton’s Tyrosine Kinase Inhibitors. N Engl J Med. Massachusetts Medical Society; 2022;386:735–43.
  - This study identified kinase-impaired mutants of BTK as a resistance mechanism to therapy.
- Montoya S, Bourcier J, Noviski M, Lu H, Thompson MC, Chirino A, et al. Kinase-impaired BTK mutations are susceptible to clinical-stage BTK and IKZF1/3 degrader NX-2127. Science. 2024;383:eadi5798.
  - This article demonstrated efficacy of BTK degradation in wild-type and specific kinase-impaired BTK mutants.
- Shadman M, Brown JR, Williams R, Mohseninejad L, Yang K, Rakonczai P, Lamanna N, Xu S, Cleary Cohen A, O’Brien SM, Tedeschi A, Tam CS. Efficacy of zanubrutinib versus acalabrutinib for relapsed or refractory chronic lymphocytic leukemia (R/R CLL): a matching-adjusted indirect comparison (MAIC). Ther Adv Med Oncol. 2025 Jul 8;17:17588359251340554.
  - This paper reports on a head-to-head trial of zanubrutinib versus acalabrutinib in R/R CLL and suggests superiority of zanubrutinib.
- Chong, S.J.F., Lai, J.X.H., Iskandar, K. et al. Superoxide-mediated phosphorylation and stabilization of Mcl-1 by AKT underlie venetoclax resistance in hematologic malignancies. *Leukemia* **39**, 2477–2491 (2025).
  - This study identifies the role of radical oxygen species in driving Mcl-1 stabilization to abrogate venetoclax efficacy.
- Liang S, Shao X, Meng X, Cui Y, Sun C, Sun J, et al. HGF/c-MET axis contributes to CLL cell survival by regulating multiple mechanisms making it a potential therapeutic target for CLL treatment. Front Pharmacol. 2025;16:1612916.
  - This study examines the impact of c-MET/HFG signaling on restructuring antiapoptotic mitochondrial reliance.

## Data Availability

No datasets were generated or analysed during the current study.
